# RJAfinder: An automated tool for quantification of responding to joint attention behaviors in autism spectrum disorder using eye tracking data

**DOI:** 10.3389/fnins.2022.915464

**Published:** 2022-11-17

**Authors:** Jie Zhang, Ziyi Li, Yige Wu, Adam Yongxin Ye, Lei Chen, Xiaoxu Yang, Qixi Wu, Liping Wei

**Affiliations:** ^1^Pharmacy Department of Beijing Chao-Yang Hospital, Capital Medical University, Beijing, China; ^2^State Key Laboratory of Protein and Plant Gene Research, Center for Bioinformatics, School of Life Sciences, Peking University, Beijing, China; ^3^School of Life Sciences, Peking University, Beijing, China

**Keywords:** autism spectrum disorder, eye tracking, joint attention, automated tool, behavior assessment

## Abstract

Deficits in responding to joint attention (RJA) are early symptoms of autism spectrum disorder (ASD). Currently, no automated tools exist for identifying and quantifying RJA behaviors. A few eye tracking studies have investigated RJA in ASD children but have produced conflicting results. In addition, little is known about the trajectory of RJA development through developmental age. Here, a new video was designed including 12 clips of an actor pointing to or looking at an object. Eye tracking technology was used to monitor RJA in three groups: 143 ASD children assessed with the Autism Diagnostic Interview-Revised (ADI-R) and the Autism Diagnostic Observation Schedule (ADOS) (4–7 years old), 113 age- and gender-matched typically developing children (TDC), and 43 typically developing adults (TDA) (19–32 years old). RJAfinder was developed in R and MATLAB to quantify RJA events from the eye tracking data. RJA events were compared among the three groups. Spearman correlation coefficients between total number of RJA events in ASD and the Social Responsiveness Scale (SRS) scores were calculated. A logistic regression model was built using the average valid sampling rate and the total number of RJA events as two predictive variables to classify ASD and TDC groups. ASD children displayed statistically significantly less RJA events than the TDC and TDA groups with medium-to-large-sized effects. ASD and TDC children both displayed more RJA events in response to pointing stimuli than to looking stimuli. Our logistic regression model predicted ASD tendency with 0.76 accuracy in the testing set. RJA ability improved more slowly between the ages of 4–7 years old in the ASD group than in the TDC group. In ASD children, RJA ability showed negative correlation with SRS total T-score as well as the scores of five subdomains. Our study provides an automated tool for quantifying RJA and insights for the study of RJA in ASD children, which may help improve ASD screening, subtyping, and behavior interventions.

## Introduction

Responding to joint attention (RJA) refers to the ability of an individual to direct their attention toward an object by following a partner’s verbal and non-verbal indications, eye gaze, or pointing, during a social interaction (Moore and Dunham, 1995). These indications can cue in a social partner as to what is going on in the other partner’s mind. It is also related to a temporal and spatial context. So RJA is a complex and dynamic process that involves coordinating attention toward a social partner and an object of mutual interest (Bakeman and Adamson, 1984; Moore and Dunham, 1995). RJA skills form a basis for the development of social cognition and language (Tomasello and Farrar, 1986; Moore and Dunham, 1995; Carpenter et al., 1998; Flom et al., 2007; Mundy and Newell, 2007; Gillespie-Lynch et al., 2015). Individuals who do not engage in RJA may display impaired social development (Bruinsma et al., 2004; Sheinkopf et al., 2004; Hahn, 2016). Children with autism spectrum disorder (ASD) often do not follow the RJA indications of others (Charman, 2003; Bruinsma et al., 2004; Dawson et al., 2004; Naber et al., 2008). RJA deficit is a primary and cardinal feature that could distinguish ASD from other developmental disorders (Mundy and Sigman, 1989).

Responding to and initiating joint attention (IJA) are two aspects and observable indicators of joint attention (JA). Because RJA is a passive action, it is much easier to identify and quantify. Eye tracking technologies began emerging as an important tool in the field to quantify attention of ASD in 2002 (Klin et al., 2002). Eye trackers could automatically record the exact spatial and temporal patterns of eye movements. Given the importance of joint attention in early development, several eye tracking studies have investigated RJA in children with ASD (Bedford et al., 2012; Navab et al., 2012; Swanson and Siller, 2013; Thorup et al., 2016; Caruana et al., 2017), but differences emerged in the results. For instance, a study by Bedford et al. (2012) found that 13-month-old children at high risk for ASD who were eventually diagnosed at 3 years old showed decreased attention to congruent objects (the object the actor’s gaze followed was the target object) compared with low-risk controls. However, a study by Swanson and Siller (2013) used eye tracking technology to measure joint attention in 21 children with ASD and 24 TDC, found no significant differences between the two groups in terms of the total gaze time allocated to targets and faces of interest under either congruent or incongruent conditions. Possible reasons for the inconsistent results include small sample sizes, imperfect stimuli, and confounding factors. Moreover, most studies mainly considered the duration of gaze in different regions after the joint attention behaviors induced, and may ignored the dynamic process of this behavior. So, measuring the participants’ eye gaze using the video stimuli with dynamic social scenes is a much better research mode. Because this type of stimuli could simulate naturalistic situations, and the participants could view the video from a first-person perspective. Also to our knowledge, no automated methods have been published for rapid identification of RJA behaviors using eye tracking technologies.

In addition, joint attention may develop with age. The acquisition of RJA skills is a major milestone in early childhood development, which typically emerges between 6 and 12 months of age and is well established by 18 months of age (Adamson and Bakeman, 1985; Brooks and Meltzoff, 2002, 2005). The trajectory for RJA development in children with ASD has been explored, but most focus on the RJA level in infants and toddlers (Tomasello, 1995; Carpenter et al., 1998; Mundy et al., 2007, 2009). The deficits in children with ASD are obvious by the age of 24 months (Presmanes et al., 2007; Naber et al., 2008; Mundy et al., 2009; Ibanez et al., 2013), and may manifest as early as 12–18 months (Osterling and Dawson, 1994; Baron-Cohen et al., 1996; Swettenham et al., 1998). Mundy et al. (2007) demonstrated that infants displayed age-related changes in JA behaviors from 9 to 18 months. A few studies have focused on joint attention ability in preschool children with ASD (Casenhiser et al., 2013; Goods et al., 2013). Some studies have reported that in individuals with ASD, IJA always showed defects from preschool to adolescence, while RJA tended to be normal gradually (Naber et al., 2008; Barbaro and Dissanayake, 2013). JA intervention improved language outcome significantly in children with autism under 5 years of age (mean 58.25 months) (Kasari et al., 2008). Defining levels of joint attention is important in determining if children are engaging in age-appropriate joint attention. Though there is now a good deal of evidence demonstrating predominant focus on the RJA level in infants and toddlers, it is not yet clear whether preschool ASD children develop to the normal level with TDC.

The current study aimed to optimize eye tracking research paradigm for RJA, to develop a tool for rapid quantification of RJA capability and deficiencies, and to evaluate the development of RJA ability in preschool autistic children. Here, we used R and MATLAB, to design the tool, RJAfinder, that combined video stimuli with automated RJA identification method. RJAfinder was used to evaluate RJA behaviors in the largest group of individuals studied to date, including children with ASD, TDC, and typically developing adults (TDA). The performance and accuracy of the tool were evaluated based on: (1) the agreement in identification of RJA behaviors by the automated tool and manual coding results, (2) the correlation between the total number of RJA events and social skills measured by the traditional Social Responsiveness Scale (SRS) (Constantino and Gruber, 2005). The hypothesis was that RJA ability measured by RJAfinder could distinguish between three groups, the ASD group was worse than the other two groups, and the TDA group performed the best in this eye tracking measurement paradigm. We predicted that RJAfinder could perform as a potential tool for the quantification of RJA deficiencies.

## Materials and methods

### Participants

The study was approved by the Peking University Institutional Review Board (No. IRB00001052-15003). Before participation, all participants or their legal guardians supplied written informed consent. All participants completed the experimental procedures.

Children with ASD were recruited from two behavior training institutions in China: Stars and Rain Educational Institute in Beijing and Elim Autism Training Institution in Tsingtao. Each participant underwent evaluation by a child psychiatrist as well as comprehensive phenotypic assessment by certified evaluators using the “gold standard” diagnostic tool: ADI-R (Lord et al., 1994). We adopted the ADI-R criteria, which was previously applied by the Simons Simplex Collection (SSC) (Fischbach and Lord, 2010), to confirm the diagnosis of ASD. A diagnosis of ASD was confirmed if a participant met any one of the following four criteria on the ADI-R: (1) exceeding standard cutoffs on both the Social and Communication domains; (2) exceeding the standard cutoff on the Social domain and within two points of the Communication cutoff; (3) exceeding the standard cutoff on the Communication domain and within two points of the Social cutoff; or (4) within one point of cutoffs for both the Social and Communication domains. Children with ASD were excluded from participating in the study if they met any one of the following conditions: (1) the ASD diagnosis was not confirmed by a child psychiatrist; (2) they did not meet the ADI-R criteria for ASD described above; or (3) parents reported vision impairments in their children, including strabismus, astigmatism, or amblyopia, during pre-screening interviews.

The TDC group was recruited from Haijun Jiguan Kindergarten in Beijing. All participants in the group were evaluated using SRS (Constantino and Gruber, 2005) to rule out autistic social impairment; participants whose total T-score was 60 or higher were excluded. TDC with vision impairments (strabismus, astigmatism, or amblyopia) reported by their parents were also excluded from the study.

The TDA group was recruited from Peking University and the National Institute of Biological Sciences, Beijing. Potential participants completed the Adult Autism Spectrum Quotient (AQ) questionnaire (Baron-Cohen et al., 2001) to rule out participants with autistic features (i.e., a total AQ score greater than 32), as well as a questionnaire designed by our research team to exclude participants with a history of any neurological disorders, psychiatric illness, or adverse pregnancy outcomes.

### A new stimulus video designed for the eye tracking experiment

**FIGURE 1:**
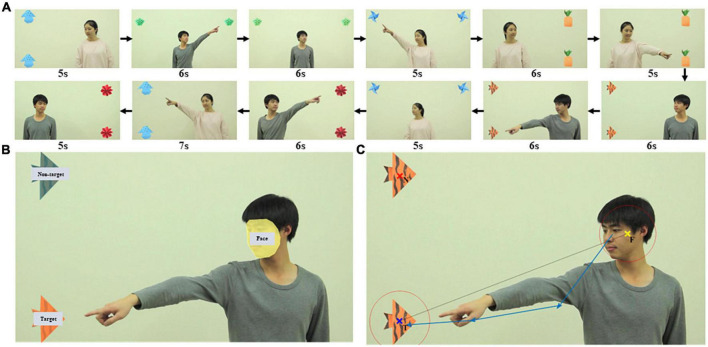
Looking and pointing stimuli used to elicit RJA of participants. **(A)** Screenshots of the 12 video clips in the order they were played, each presenting an actor who used looking or pointing to alert participants to target objects. **(B)** AOI drawn in one video clip as an example. There were three AOI groups: face, target, and non-target. **(C)** An example of an RJA event identified. The yellow, blue, and red x indicated the center of the actor’s face (F), the center of the target object (T), and the center of the non-target object (N), respectively. Two red circles were centered at F and T and the radius of each circle was 1.2 times the radius of the respective AOI. The sequence of blue vectors was a real example of a complex RJA recognized by RJAfinder with four sequential fixations and three vectors.

We designed and created a video of 12 clips, each presenting an actor who either looked at (eye-gaze shifting) or pointed to (gesture shifting) a target object to alert participants to the object’s location. We tried to minimize any confounding factors as follows. We used a male and a female actor in different clips. In each clip, the position of the actors and the objects were counterbalanced within the task to control for participants’ visual attention staying on one side or the other. Two objects (one target and one non-target) were positioned at the left, right, or top corners of the screen and each screen arrangement was presented in 4 of the 12 clips (Figure 1A). In each clip, the actor was positioned at the opposite side of the screen from the objects or in the middle of the screen when objects appeared at the top corners (Supplementary material 1 Figure 1). All non-essential objects and events that might distract a viewer’s attention from the social action were removed from the video clips. In each video clip, there was an actor who used eyes or gestures to alert participants to look at the target object. The actor looked straight ahead for the first 2 seconds. Then the actor performed the action accordingly and maintained it for the next 2 seconds. Finally, the actor went back to the starting state to look straight in the last 2 seconds. The clips ranged in length from 5 to 7 seconds (Figure 1A). Each clip was played immediately after the previous one. The video was shown at full-screen and 1,920 × 1,080 pixels resolution. Rate of presentation was 30 frames/s. The video clips are available as Supplementary material 2. The meaning of the video clip names was explained in Supplementary material 3.

### Experimental procedure and setting

Participants sat in a chair at a distance of 65 cm from the screen. A brief calibration routine was conducted in which participants looked at a series of five points on the screen. The eye tracking procedure was started only after at least four points were marked as correctly calibrated for each eye. Video play and data recording began after successful calibration.

A dark pupil tracking method was used to collect eye tracking data, with the hardware and software created by Tobii, Inc. (accuracy ± 0.4°, precision ± 0.15° over a ± 35° horizontal and vertical range). The system was integrated into the screen setup, with the Tobii TX300 eye tracker placed below the 23-inch screen.

### Eye tracking data acquisition

Eye tracking data were recorded at a frequency of 300 Hz (one sampling point every 3.33 ms). Fixations were classified from the raw data by applying the I-VT filter (classifier: 30 degrees/s, Velocity calculator window length: 20 ms), that was the default fixation filters setting in the software (Tobii Studio 3.3.1). Data points with angular velocity below the threshold value (30 degrees/s) were classified as “fixation” and data points above the threshold were classified as “saccade.” The output raw data were based on the average movements of both eyes (Supplementary material 1 Table 1). Gap-filling was applied. Noise reduction was not applied. Eye-tracking data with coordinates and time information were exported from Tobii Studio in a table format.

The valid sampling rate was defined as the proportion of sampling points in which the eye tracker successfully detected the pupils of both eyes among all sampling points in each clip. A low valid sampling rate indicated that the participant did not focus on the screen or had swung his/her head. To ensure the integrity of eye tracking data used for analysis, only participants whose average valid sampling rate was above 60% were used in subsequent analyses.

### Development of RJAfinder, an automated tool to detect RJA

For each video clip, the areas of interest (AOI) were drawn surrounding the actor’s face, the target object, and the non-target object in Tobii Studio (Figure 1B). The coordinates of the center of the actor’s face (F), the center of the target object (T), and the center of the non-target object (N) were defined as the arithmetic mean value of the “X, Y” coordinates of all points within each AOI. The radius of the AOI (R_*F*_, R_*T*_, and R_*N*_, respectively) was defined as the maximum distance from the center of the AOI to its edge. The vector from F to T was defined as the F-T benchmark vector (Figure 1C).

We developed RJAfinder, an automated tool, to extract RJA from filtered eye tracking data. The tool was implemented in both R (version 3.4.1) (R Core Team, 2018) and MATLAB (MATLAB and Statistics Toolbox Release 2017a, The MathWorks, Inc., Natick, Massachusetts, United States). RJAfinder used the eye-tracking data from Tobii Studio in a table format as input and then extracted all fixations between the start and end of the actor’s action. It then constructed fixation vectors that were the directional vectors between two consecutive fixations. A fixation sequencing was a sequence of consecutive fixation vectors. RJAfinder considered a fixation sequence to be an RJA event if the following four criteria were satisfied: (1) the distance from the first fixation in the sequence to F was less than 1.2 times R_*F*_; (2) the distance from the last fixation in the sequence to T was less than 1.2 times R_*T*_; (3) the distance from the fixation (except the first one) to T was less than the distance to N; (4) all the intersection angles between the fixation vectors in the sequence and the F-T benchmark vector were less than 90°. An example of an RJA event identified by RJAfinder is presented in Figure 1C. We made the R script of RJAfinder freely available as Supplementary material 4.

### Evaluation of RJAfinder’s performance and accuracy in identifying RJA behaviors

We evaluated the accuracy of RJAfinder as follows:

1. **Agreement with manual coding results.** We constructed a test set by randomly selecting 50 video clips of looking stimuli and 50 video clips of pointing stimuli. Two researchers who were familiar with RJA behaviors watched the video clips and independently identified the occurrence or absence of RJA events in each video clip. An RJA event would be identified by the researcher if the gaze fixation of the participant started from the actor’s face after the looking or pointing action, and ended at the target object before the actor’s action ended. The inter-rater agreement between the existence of RJA events identified by RJAfinder and two researchers was calculated using the percentage of consistency and the Cohen’s kappa statistic (Cohen, 1960; Hallgren, 2012).
2. **Correlation with SRS assessment.** Spearman correlations were used to explore the relationship between the total number of RJA events and social ability (measured by total SRS T-scores) of each participant in the ASD and TDC groups. Benjamini-Hochberg false discovery rate procedure was applied for the multiple comparison correction.

### Comparison of RJA ability among the three study groups

The Mann–Whitney *U*-test was used to analyze the proportion of participants in three groups that exhibited RJA events while watching the 12 clips. Further, we used two values to quantify a participant’s eye tracking behaviors: the average valid sampling rate of eye tracking and the total number of RJA events extracted by RJAfinder among video clips in each participant. Mann–Whitney *U*-tests were used to test differences among the three groups in terms of average valid sampling rate and total number of RJA events for looking clips, pointing clips, and all 12 clips together.

### Predictive ability of RJAfinder classification model

A logistic regression model using the average valid sampling rate and the total number of RJA events as two predictive variables was built to classify whether a participant had ASD. The dataset of 143 ASD and 113 TDC was randomly split into 80% as a training set (114 ASD and 90 TDC) and 20% as a testing set (29 ASD and 23 TDC). The logistic regression model was fitted on the training set. Cross-validation was also implemented. 10-fold cross-validation was used to divide the training set into two parts: CV_train and CV. The model was trained with CV_train dataset and tested with CV dataset. To examine the goodness of fit of the model, we examined linearity assumption, influential values, and multicollinearity. The two predictive variables (the average valid sampling rate and the total number of RJA events) were both quite linearly associated with ASD outcome in logit scale (Supplementary material 1 Figure 2A). There were no influential observations, because Cook’s distance was less than 0.1 and no data points had standardized residual larger than 3 (Supplementary material 1 Figure 2B). Also, there was no strong collinearity, because the variance-inflation factor (VIF) of both predictive variables was 1.03, well below 5. These demonstrated that the model was appropriate.

The receiver operating characteristic curve (ROC) and area under curve (AUC) were calculated based on the testing set. A mosaic plot, in which the area was proportional to the sample size, was plotted to show the relationship between the proportion of confirmed ASD cases and those predicted by the model. In addition, Spearman correlation coefficients were calculated and statistically tested for SRS T-scores and the probability was predicted (among test/ASD). Benjamini-Hochberg false discovery rate procedure was applied for the multiple comparison correction. All model fitting and statistical testing were performed using the R environment (version 3.4.1) (R Core Team, 2018) with additional packages, caret (Kuhn, 2008) and pROC (Robin et al., 2011).

Statistical computations were conducted in R (version 3.4.1) (R Core Team, 2018) with an alpha level of 0.05, unless otherwise stated.

### Progression of response to looking and pointing RJA indications with age

Linear regression analysis was used to explore the development of RJA abilities with respect to age in ASD and TDC groups from 4 to 7 years old.

## Results

### The characteristics of participants in the three groups

We recruited an initial group of 203 children with ASD (4–7 years old), 137 TDC (4–7 years old), and 52 TDA (19–32 years old) as three independent groups. After further screening, the group of study participants was narrowed down to 167 children with ASD, 116 TDC, and 44 TDA. After excluding the participants whose data with the average sampling rate less than 60%, the final group of participants included 143 ASD, 113 TDC, and 43 TDA (Supplementary material 1 Figure 3). A summary of participant characteristics and scores on the questionnaires is shown in Table 1. The details of the participant characteristics are shown in Supplementary material 3. TDC participants matched ASD participants in chronological age (Mann–Whitney *U*-test, *p* = 0.12) and gender (Fisher’s exact test, *p* = 0.12). The SRS T-Scores of ASD participants were significantly higher than those of TDC (Mann–Whitney *U*-test, *p* < 2.2 × 10^–16^) (Supplementary material 1 Figure 4).

**TABLE 1:** Summary of participant characteristics in the three groups.

Characteristics	ASD (*n* = 143)	TDC (*n* = 113)	TDA (*n* = 43)	*P*-value
Male gender, no. (%)	126 (88)	91 (81)	20 (47)	0.12
Age (SD) [range], y	5.37 (0.74) [4.0–6.8]	5.54 (0.75) [4.0–6.8]	25.44 (2.62) [19.2–31.4]	0.12
SRS T-score (SD) [range]	76.44 (12.50) [43–108]	48.73 (5.67) [36–59]	−	<0.001***
ADI-R score (SD) [range]				
Reciprocal social interaction	19 (5.0) [8–29]	−	−	−
Communication				−
Non-verbal	10.9 (2.7) [5–14]			
Verbal	16.1 (3.4) [7–23]			
Repetitive behaviors	5.0 (2.5) [0–11]			−
Diagnosis at or before 36 months	4.1 (1.1) [1–5]			
AQ score (SD) [range]	−	−	19.23 (6.10) [9–31]	

***Indicates significant difference (*p* < 0.001).

### RJA detected by RJAfinder

Using RJAfinder, we extracted all RJA events for each participant in each video clip. The number of RJA events identified by RJAfinder for each participant in each clip is shown in Supplementary material 3. To illustrate this, a TDC participant’s RJA is shown as an example in Supplementary material 1 Figure 5.

### Evaluation of RJAfinder

1. **Agreement with manual coding results.** The two researchers showed 92% (46/50, Cohen’s kappa = 0.839) consistency with each other in identifying RJA in the looking video clips and 94% (47/50, Cohen’s kappa = 0.819) consistency in the pointing video clips. Among the video clips where the two researchers showed consensus, RJAfinder showed 100% (46/46, Cohen’s kappa = 1) consistency with the consensus in the looking video clips and 85% (40/47, Cohen’s kappa = 0.603) consistency in the pointing video clips (Table 2). The range of Kappa calculation values is −1∼1, and usually falls in 0∼1. Different values represent different levels of agreement (Supplementary material 1 Table 2; Landis and Koch, 1977). These results indicated that RJAfinder was highly consistent with the manual coding results in looking videos and moderately consistent in pointing videos, which demonstrated the accuracy of RJAfinder’s ability to identify RJA.

**TABLE 2:** The agreement of the two researchers with RJAfinder.

	Looking videos		Pointing videos	
		
	Cohen’s kappa	Consistency	Cohen’s kappa	Consistency
R1 with R2	0.839	92% (46/50)	0.819	94% (47/50)
R1 with RJAfinder	0.960	98% (49/50)	0.610	84% (42/50)
R2 with RJAfinder	0.879	94% (47/50)	0.554	82% (41/50)
Same part of R1 and R2 with RJAfinder	1.000	100% (46/46)	0.603	85% (40/47)

R1, Researcher 1; R2, Researcher 2.

Then, we reviewed the video clips with the inconsistent number of RJA events identified by RJAfinder and manual coders, and summarized reasons for the differences. One RJA event in one of the pointing video clips was identified by RJAfinder but not by the researchers who, upon examining the RJA event detected by RJAfinder, concluded that RJAfinder was correct (Supplementary material 1 Figure 6A). In one looking clip and four pointing clips, RJAfinder did not identify the RJA event because the position of the fixations exceeded 1.2 times the diameter of the actor’s face or target (Supplementary material 1 Figure 6B). In one looking clip and two pointing clips, RJAfinder did not identify the RJA events because some fixation vectors within the sequence returned to the actor (Supplementary material 1 Figure 6C). These results showed that RJAfinder followed the definition of RJA events in this study more strictly than the human researchers did.

2. **Correlation of RJA with SRS scores in ASD and TDC groups.** We compared the total number of RJA events with social skills measured by SRS (Supplementary material 1 Table 3). Although none of the correlation analyses were significant after Benjamini-Hochberg false discovery rate correction, the total number of RJA events showed negative correlation with SRS total T-score as well as the scores of five subdomains in the ASD group. This suggested that the children with ASD who were more severe in the social defect measured by SRS could exhibit less numbers of RJA events, and RJA ability measured by RJAfinder reflected the social communication deficits of children with ASD.

### Comparison of RJA ability among the three study groups

**FIGURE 2:**
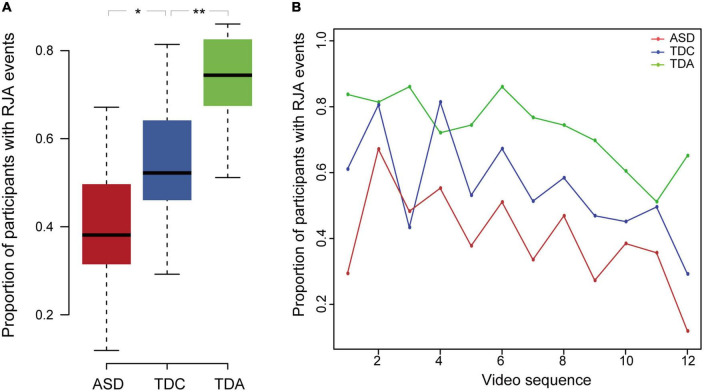
The proportion of participants with RJA events differed among three groups. **(A)** The proportion of participants having RJA events in the three groups for the 12 clips. Error bars represented standard error from the mean. **Indicates significant differences (*p* < 0.01), *indicates significant differences (*p* < 0.05). **(B)** Proportion of RJA events observed in the ASD, TDC, and TDA groups during all 12 video clips. In all three groups, proportion of participants with RJA events was lower for video clips shown late in the sequence than for clips shown early in the sequence.

The average proportion of participants having one or more RJA events in all video clips in the TDC group was significantly higher than that in the ASD group (Mann–Whitney *U*-test, *p* = 2.4 × 10^–2^, effect size *r*-value = 0.46) and significantly lower than that in the TDA group (Mann–Whitney *U*-test, *p* = 7.2 × 10^–3^, effect size *r*-value = 0.55) (Figure 2A and Supplementary material 1 Table 4). It showed a medium to large effect size. All three groups displayed a decreasing ability to respond to RJA stimuli in video clips shown later in the sequence (Figure 2B). The proportion of ASD, TDC, and TDA participants who exhibited RJA ranged from 11% to 67%, 29% to 81%, and 51% to 86%, respectively, in the 12 clips. Within both the ASD and TDC groups, the proportion of participants with RJA was lower for the looking stimuli than for the pointing stimuli (Supplementary material 1 Figure 7). The RJA ability displayed by children with ASD in response to the pointing clips was similar to that of the TDC in response to the looking clips. In contrast, the proportion of TDA participants who reacted to stimuli was similar for both the looking and pointing clips over time.

**FIGURE 3:**
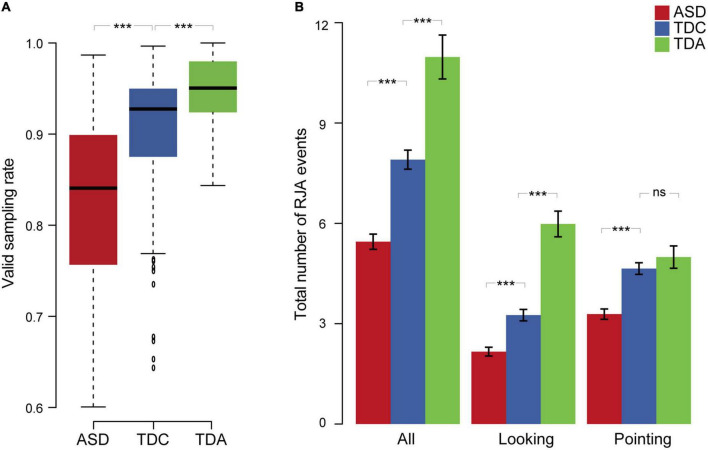
Eye tracking behaviors and RJA events among three groups. **(A)** The valid sampling rate of the three groups for 12 clips. **(B)** Total number of RJA events observed in the three groups for 12 clips. Data are presented as mean ± SEM. Error bars represent standard error from the mean. ***Indicates significant differences (*p* < 0.001), “ns” indicates a non-significant difference.

We compared the different groups in terms of the valid sampling rate and the total number of RJA events. The valid sampling rate in the TDC group was significantly higher than that in the ASD group (Mann–Whitney *U*-test, *p* = 1.95 × 10^–11^, effect size *r*-value = 0.42) and significantly lower than that in the TDA group (Mann–Whitney *U*-test, *p* = 1.93 × 10^–4^, effect size *r*-value = 0.30) (Figure 3A and Supplementary material 1 Table 4). The total number of RJA events in the ASD group was significantly lower than that in the TDC group (Mann–Whitney *U*-test, *p* = 4.91 × 10^–10^, effect size *r*-value = 0.39) for both the looking clips (Mann–Whitney *U*-test, *p* = 1.05 × 10^–6^, effect size *r*-value = 0.31) and the pointing clips (Mann–Whitney *U*-test, *p* = 2.06 × 10^–8^, effect size *r*-value = 0.35). The total number of RJA events in the TDC group was significantly lower than that in the TDA group for both sets of clips combined (Mann–Whitney *U*-test, *p* = 1.84 × 10^–5^, effect size *r*-value = 0.34) and for the looking clips (Mann–Whitney *U*-test, *p* = 3.14 × 10^–9^, effect size *r*-value = 0.47), but not for the pointing clips (Mann–Whitney *U*-test, *p* = 3.46 × 10^–1^, effect size *r*-value = 0.08) (Figure 3B and Supplementary material 1 Table 4). Thus, the total number of RJA events elicited medium-sized effects between ASD and TDC and medium-to-large-sized effects between ASD and TDA.

Comparing RJA behavior induced by looking and pointing stimuli, all participants including children with ASD had more total number of RJA events induced by pointing stimuli than that induced by looking stimuli. This result indicated that the TDC’s level in pointing induced RJA behavior was close to that of normal adults. But they have not developed the ability to the same level with normal adults in looking-induced RJA behavior. However, children with ASD have developmental retardation in both looking- and pointing-induced RJA behaviors. There were no significant differences in responses to stimuli between boys and girls within either the ASD or TDC groups (Supplementary material 1 Figure 8).

### Logistic regression model could distinguish between ASD and TDC groups

**FIGURE 4:**
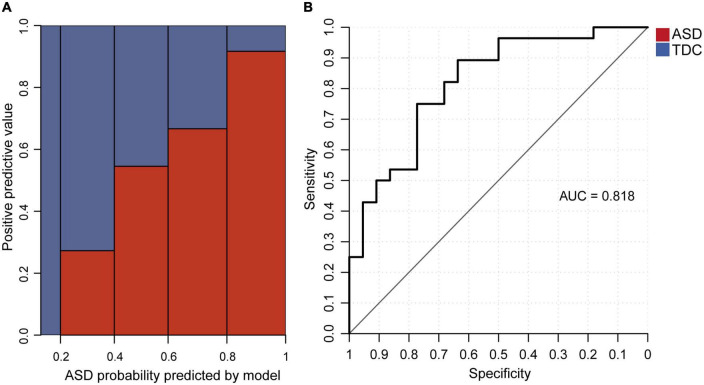
The logistic regression model for distinguishing between ASD and TDC. **(A)** Relationship between the proportion of verified ASD participants and the ASD probability predicted by the logistic regression model in the testing set. **(B)** AUC of the logistic regression model in the testing data set.

We investigated whether the core features derived from the eye tracking data could distinguish between ASD and TDC. We used the average valid sampling rate and the total number of RJA events as two predictive variables and built a logistic regression model to predict whether a participant had ASD. Coefficients of the logistic regression model are shown (Supplementary material 1 Table 5). The accuracy on the 80% training set and the 20% testing set was 0.709 and 0.76, respectively (Supplementary material 1 Figure 9A). The mean and standard deviation of the 10 CV datasets using the 10-fold cross-validation on the 80% training set were shown in Supplementary material 1 Table 6. The mean and standard deviation of accuracy were 0.74 and 0.12. The positive-predictive value increased with the ASD probability predicted by the model (Figure 4A). The AUC of the model was 0.818 (Figure 4B). The regression coefficients were statistically significant (*p* = 1.1 × 10^–4^ for average valid sampling rate and *p* = 2.8 × 10^–4^ for the total number of RJA events). When the valid sampling rate decreased by 0.1, the odds ratio predicting ASD increased by 2.29-fold, and one additional RJA event from the 12 video clips led to a 1.24-fold odds ratio predicting that a participant was a TDC.

We also examined the Spearman correlation coefficients between SRS T-scores and the ASD probability predicted by the model. Among the testing set, SRS T-scores for the subdomains of Social Awareness, Social Cognition, Social Communication, Social Motivation, and Autistic Mannerisms were all significantly and positively correlated with the predicted ASD probability (*p* < 0.01, Supplementary material 1 Table 7). As a result, the SRS total T-score was significantly and positively correlated with the ASD probability predicted by the model (Benjamini-Hochberg corrected *p* = 1.06 × 10^–14^). The correlation between the SRS total T-score and the predicted ASD probability was still positive and significant within the ASD group (Benjamini-Hochberg corrected *p* = 0.0276), but showed no significant correlation within the TDC group (Benjamini-Hochberg corrected *p* = 0.83) (Supplementary material 1 Figure 9B and Supplementary material 1 Table 7). This suggests that our model could be used to distinguish children with ASD from TDC and to predict the severity of social behavior deficits.

### Development of looking and pointing RJA ability with age in ASD and TDC participants

**FIGURE 5:**
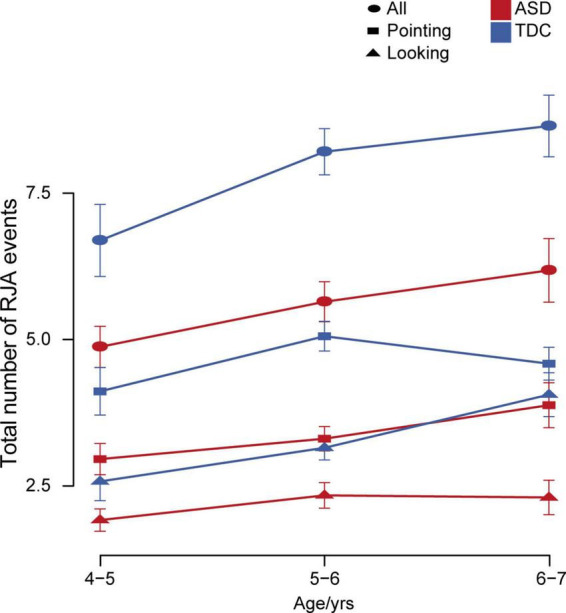
Changes in total number of RJA events with age in the ASD and TDC groups. Error bars represent standard error from the mean.

We examined differences in the total number of RJA events at different ages. Linear regression analysis showed that age was significantly positively associated with the total number of RJA events in both the ASD and TDC groups, but the TDC group showed a larger increase over the same age span (ASD: *p* = 0.0070; TDC: *p* = 0.0059). In the ASD group, response to the pointing stimuli, but not the looking stimuli, had a significant positive correlation with age (*p* = 0.010). In contrast, in the TDC group, response to the looking stimuli, but not the pointing stimuli, had a significant positive correlation with age (*p* = 0.0025). The gap in RJA ability in response to pointing vs. looking stimuli increased in the ASD group with age, but decreased in the TDC group (Figure 5). There was no statistical significance between TDA participants and TDC participants aged 4–5 years (Mann–Whitney *U*-test, *p* = 0.078), 5–6 years (Mann–Whitney *U*-test, *p* = 0.98), or 6–7 years (Mann–Whitney *U*-test, *p* = 0.31). The above results indicated that at the age of 4 years old, the TDC group and the TDA group had similar RJA levels induced by pointing, while the children with ASD at the age of 4–7 years old had more delayed and slower RJA ability induced by looking than that induced by pointing, and there was no significant positive correlation between age and the total number of RJA events induced by pointing.

## Discussion

In this study, RJAfinder was developed as an automated tool to detect and quantify RJA events from eye tracking data. Using this tool, we found that children with ASD demonstrated a statistically significant less RJA events than the normal control groups with medium-to-large-sized effects. A logistic regression model was built using the RJA features and could classify ASD with 0.76 accuracy. In addition, we reported that RJA ability increased more slowly between the ages of 4–7 in children with ASD than in TDC. These results suggest that children with ASD are developmentally delayed in RJA abilities, and should be given more intervention on RJA skills before 7 years old.

Several studies have focused on detecting RJA deficits in ASD through eye tracking (Bedford et al., 2012; Falck-Ytter et al., 2012; Swanson and Siller, 2013; Thorup et al., 2016; Caruana et al., 2017; Franchini et al., 2017), but the results are controversial. Previous studies lack a solid eye tracking paradigm. We optimized the video stimuli inducing RJA behaviors in our study, including real-world stimuli and care taken to control confounding factors, and could detect gaze following joint attention as well as pointing following behaviors. To minimize artifacts in our experiments, we took great care to create video stimuli that included actors of both genders, varying locations of the objects, and clean backgrounds. The side of the screen where the target and non-target objects were presented alternated among videos to avoid visual perseveration from influencing the location of the participants’ first fixations. In conclusion, this new approach to study RJA appears very promising because it reveals the temporal and spatial dynamics of this RJA behavior and not simply the likelihood of its occurrence.

To our knowledge, our study tested the largest sample set of any previous study on eye tracking for determination of RJA in ASD individuals, gender- and age-matched TDC, and TDA. All ASD participants were assessed with ADI-R (Lord et al., 1994) by credentialled evaluators to confirm the clinical diagnosis. All TDC participants were gender- and age-matched with the ASD group and those with social behavior deficits assessed by SRS (Constantino and Gruber, 2005) were ruled out. The visual problem was another important exclusion indicator to remove the interfering factors for the eye tracking experiment. Participants who reported strabismus, astigmatism, amblyopia or other visual problems when doing the medical history questionnaire by their parents or themselves were ruled out. To ensure the validity of our statistical analyses, we excluded participants with the valid eye tracking sampling rate less than 60%. Some ASD children had difficulties in focusing on the screen and in cooperating to complete the experiment. So inevitably a group of participants with relatively low cooperation were excluded. They might show more severe social ability deficits compared with those ASD children included.

On the other hand, we didn’t recruit non-ASD special children, like those with developmental delay (DD). Thus we didn’t know how they perform on RJA and if we could use RJAfinder to further distinguish between ASD and non-ASD special children. Also, some children with other psychiatric comorbidities may have been excluded in our study and remained to be investigated in future studies. Therefore the participant selection may limit our understanding of the applicability of outcomes to real specific clinical contexts-of-use. It is necessary to dig deeply into the heterogeneity of this behavior through the application of tools like RJAfinder to get a better picture of the spectrum.

In terms of analysis methods, assessing RJA by eye tracking measures is a good starting point in investigations of ASD social behaviors, that is faster and easier than parent reports or behavior observation and assessment by a professional evaluator. We designed the automated tool, RJAfinder, to track behavioral responses to stimuli quantitatively. Previous studies have mostly used the gaze duration or time to first fixation on the AOI to analyze RJA behaviors (Falck-Ytter et al., 2012; Swanson et al., 2013). These two traditional indices could reflect the interest degree of the participants’ attention, but had some limitations in showing the dynamic process of RJA behaviors. In the current study, we chose the valid sampling rate and the total number of RJA events extracted by RJAfinder as two features to quantify the RJA performance. The valid sampling rate could reflect the participants’ ability to pay attention to the stimuli on the screen. The RJA events extracted by RJAfinder could present the gaze shift, that is a dynamic movement process. The total number of RJA events could reflect the overall level of the RJA behaviors. So the application of these two values is a relatively novel entry point in this research field.

The quantification of RJA deficits is a predictor for the social deficits of ASD assessed by SRS. That said, the measures included in the current study are thought to be sensitive to the assessment of ASD social behaviors. In other words, the better a child perform RJA, the better social behaviors we can expect him/her to express. This eye-tracking paradigm measuring RJA numbers represents a promising tool for measuring social cognition and screening ASD tendency in preschoolers.

We hypothesized that TDA performed at the ceiling in this eye tracking measurement paradigm. And the results really confirmed this. From our data, around 75% of participants in TDA group showed RJA events, and average 11 RJA events were observed in TDA group for 12 video clips. Although there were differences between the TDC and TDA groups’ performance in looking clips, it was very interesting to find that there was no significant difference for the pointing clips between the two TD groups. It indicated that the RJA ability responding to pointing or other gestures could be established before 7 years old. That’s why we didn’t compare TDA group and ASD group directly in this study. But we still hoped that the results made from the comparison between TDA and TDC group, such as the difference between the performance in terms of the looking video clips and pointing video clips, could provide some clues of the development of RJA ability.

As for the development of RJA behaviors in preschool children, joint attention skills deficient displayed during early development is a potential predictor for ASD (Moore and Dunham, 1995; Carpenter et al., 1998; Adamson et al., 2017). Our results suggested that the TDC group had achieved similar levels of pointing-induced RJA as the TDA group. Meanwhile, children with ASD were developmentally delayed in both looking and pointing-induced RJA behaviors. And the looking-induced RJA behavior developed more slowly than the pointing-induced RJA during 4–7 years old. The autistic participants in this group were quite young. Thus, we cannot be certain about the joint attention and social skills these children will go on to develop. Given the age range of the ASD children studied, replication will be necessary to confirm that this pattern will hold in the broader age range in ASD population, like in toddlers, teenagers and adults. Even if the current study points to a relationship between joint attention and social abilities in preschoolers with ASD, our cross-sectional design does not allow us to draw conclusions about causality, which needs to be addressed through a longitudinal study.

Furthermore, reduced RJA in autism appears to be related to social-communicative impairment, making it an important objective for young participants benefitting from intervention programs (e.g., in interventions based on social engagement). Applied behavior analysis (ABA) is considered as the gold standard for treating children with ASD. It is essential to engage children with ASD in early intervention that uses principles of ABA for JA behaviors before the age of 7 years old. RJAfinder could compensate for other behavioral assessments during the ABA training. Through RJAfinder screening, we could assess the level of RJA development much more pertinently prior to the behavior training. Another role of RJAfinder is that we could assess the RJA skills before and after the behavioral interventions to calculate the training effects. That’s what RJAfinder could contribute to the behavior training. The question remains whether, after intervention, the JA ability of children with ASD will keep lower than that of the TDC group or could develop to the same level. We assume that more RJA numbers detected by RJAfinder predicts a better clinical outcome, which should be studied in a longitudinal examination of RJA development and the participants’ outcomes.

Joint attention has been defined both narrowly and broadly in the literatures. In other words, there are three categories of joint attention: triadic, dyadic, and shared gaze. The triadic joint attention is the broadly defined joint attention, and is the highest grade. Individuals who engage in triadic joint attention must understand both gaze and intention to establish common reference. In the narrower definition, the term joint attention refers to “looking where someone else is looking” (Sigman and Kasari, 1995). Strictly speaking, the RJA behavior identified in this study represented the relatively narrowly defined RJA, that is shared attention or gaze following behavior. Shared gaze occurs when two individuals are looking at an object, which is relatively lower grade of joint attention. While RJAfinder could also identify the three-point gaze shift by adding the vector from the target to the actor’s face. So, this tool still could be used as a potential screening tool for evaluating the phenotype severity and predicting the ASD tendency.

## Conclusion

In conclusion, the observations from this study indicate that RJAfinder could be used as a potential tool for the screening and quantification of RJA deficiencies in preschool children. Our assessment of RJA could assist ASD clinical evaluation of phenotype severity. This study could shed new light on clinical subtyping and risk prediction, and help promote individualized interventions for ASD patients.

## Data availability statement

The original contributions presented in this study are included in the article/Supplementary material, further inquiries can be directed to the corresponding author/s.

## Ethics statement

The studies involving human participants were reviewed and approved by the Peking University Institutional Review Board. Written informed consent to participate in this study was provided by the participants’ legal guardian/next of kin. Written informed consent was obtained from the individual(s) for the publication of any potentially identifiable images or data included in this article.

## Author contributions

LW designed and coordinated the study. JZ participated in the design of the eye tracking test, the design of RJAfinder, collection and analysis of phenotypic data and eye tracking data, and writing and revision of the manuscript. ZL, YW, and LC participated in the participant recruitment and eye tracking experiment. ZL and YW participated in the development of the MATLAB and R program and the data analysis. AY performed the model prediction. XY and QW participated in the data statistical analysis. All authors reviewed and approved by the final manuscript.
