# Supplementary material for: RJAfinder: An automated tool for quantification of responding to joint attention behaviors in autism spectrum disorder using eye tracking data
Source: Front Neurosci. 2022 Nov 17;16:915464. doi: 10.3389/fnins.2022.915464 (PMC9714660; doi:10.3389/fnins.2022.915464)
Supplement: Supplementary file 1 [file Data_Sheet_1.zip › Supplementary material 1-Supplementary figures and tables.DOCX]

Supplementary Material

**Supplementary Figures**

| **Target objects:**  **screen placement** | **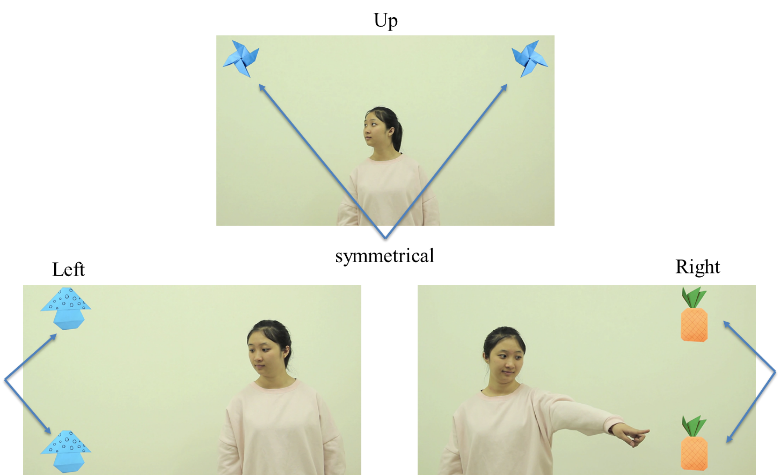** |
| --- | --- |
| **Actors: gender and direction of RJA indication** | **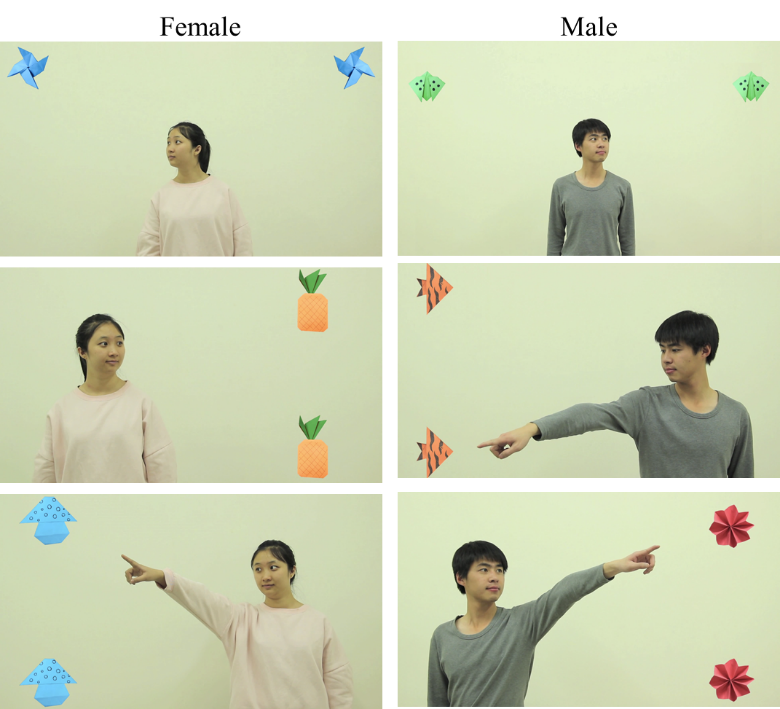** |
| **Type of RJA indication:**  **pointing or looking** | 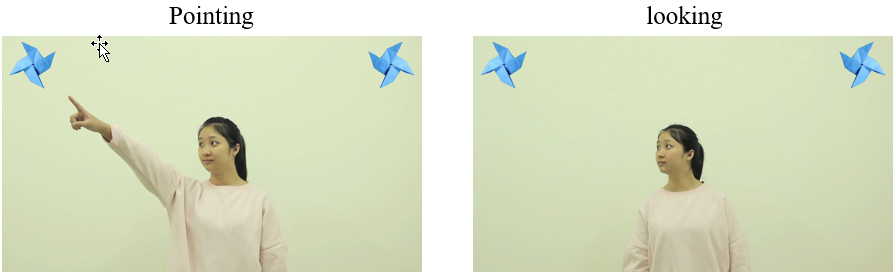 |

**Supplementary Figure 1.** Stimulus design showing balanced factors of female or male actor and screen placement of the target object. The 12 video clips were balanced to minimize confounding factors. The target and non-target objects were positioned at the left (one up and one down), right (one up and one down), or top (one left and one right) of the screen, each with 4 videos. The actor was male or female in 6 videos each. Two types of RJA indication were used: pointing and looking.


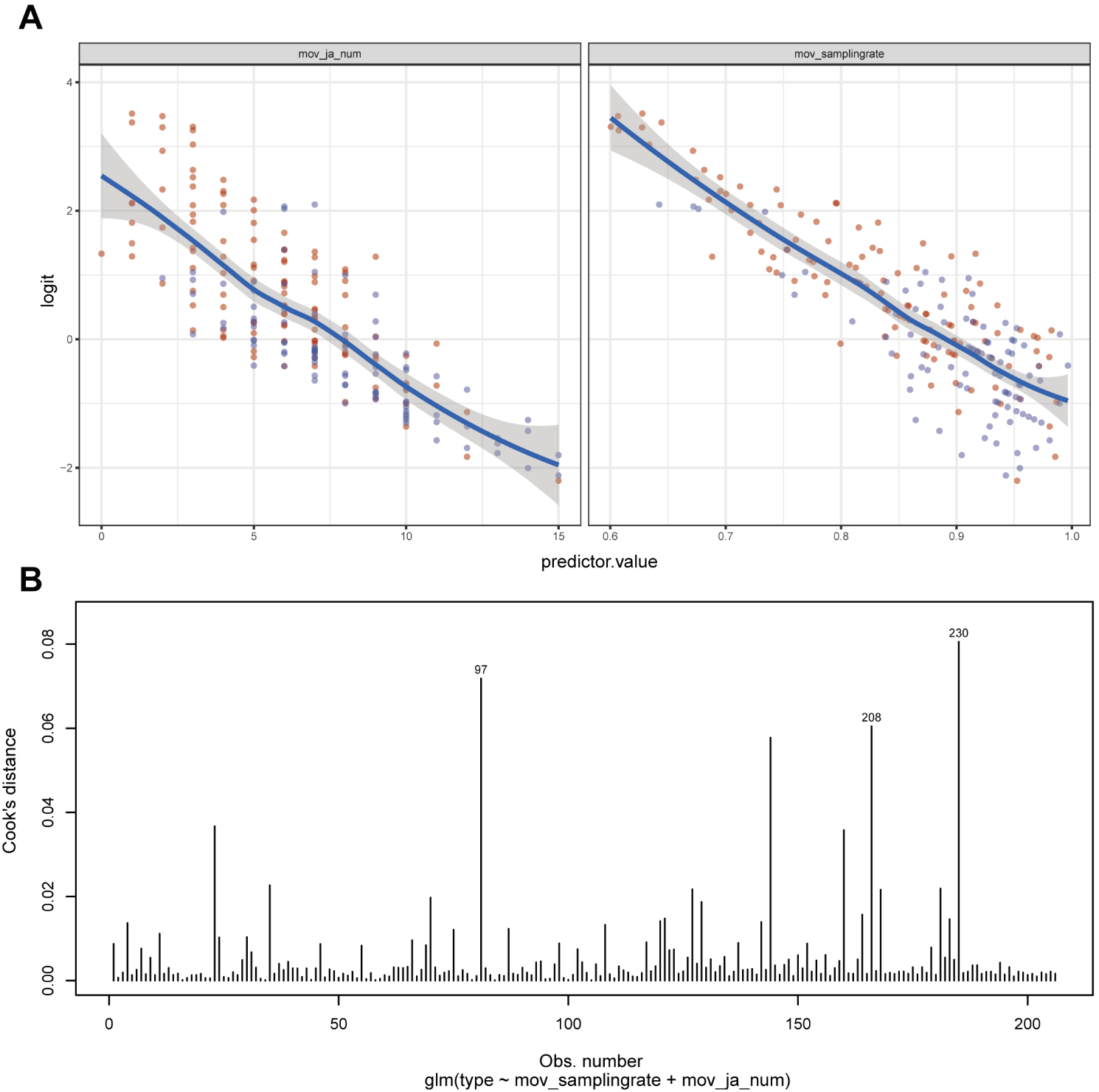


**Supplementary Figure 2.** Distributional assumptions of the measures assessed for the logistic regression. **(A)** Linearity assumption of the model. The two predictive variables (the average valid sampling rate and the total number of RJA events) were both quite linearly associated with ASD outcome in logit scale. **(B)** Influential values of the model. Cook's distance was less than 0.1 and no data points had standardized residual larger than 3.

**
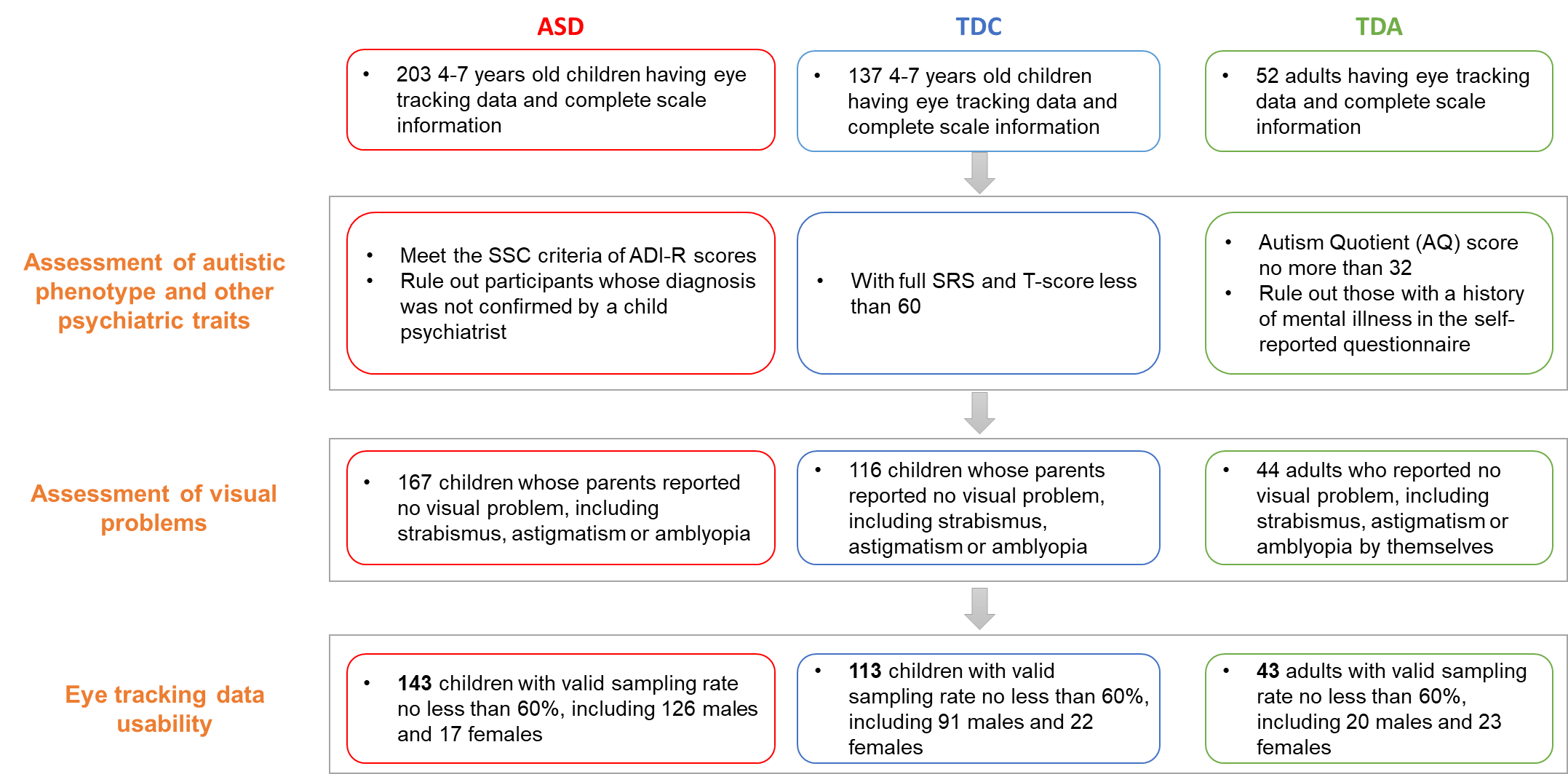
**

**Supplementary Figure 3.** Inclusion criteria and participant numbers. All participants in the ASD, TDC, and TDA groups were selected after three screening steps: assessment of autistic phenotype and other psychiatric traits, assessment of visual problems, and eye tracking data usability. 143 ASD children (126 males and 17 females), 113 TDC (91 males and 22 females), and 43 TDA (20 males and 23 females) were included in the subsequent RJA analysis.


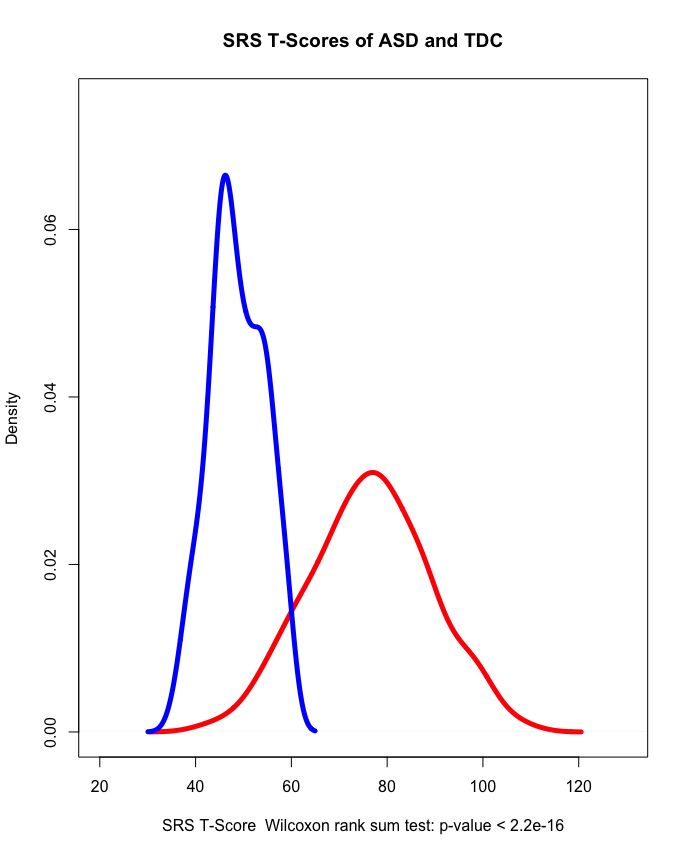


**Supplementary Figure 4.** The SRS total T-Score distribution of ASD and TDC participants. Only TDC children whose SRS T-Scores were less than 60 were included. ASD children were not selected based on their SRS T-Scores. The SRS T-Scores of ASD participants (red line) were significantly higher than those of the TDC (blue line) (Mann–Whitney U test, *p* < 2.2×10^-16^).


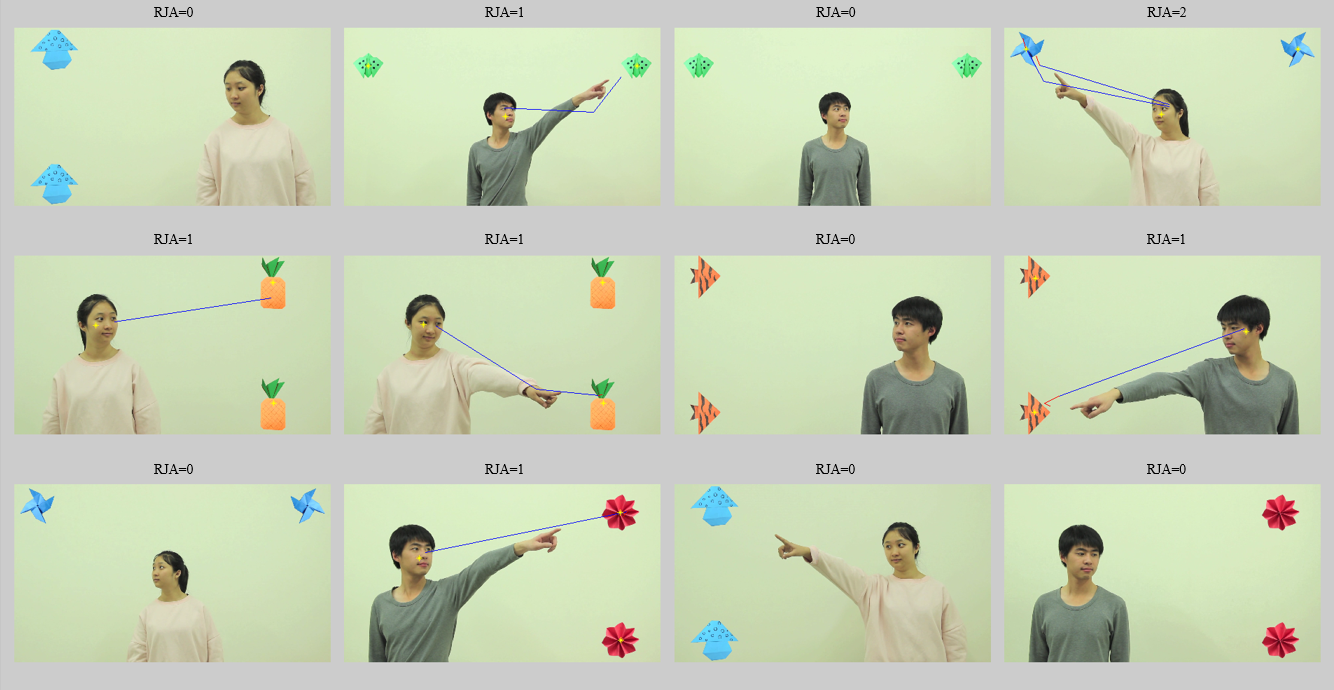


**Supplementary Figure 5.** An example of the RJA of a TDC participant extracted by RJAfinder from 12 video clips. The numbers of RJA events in each clip are shown. Blue lines indicate the RJA vectors detected.


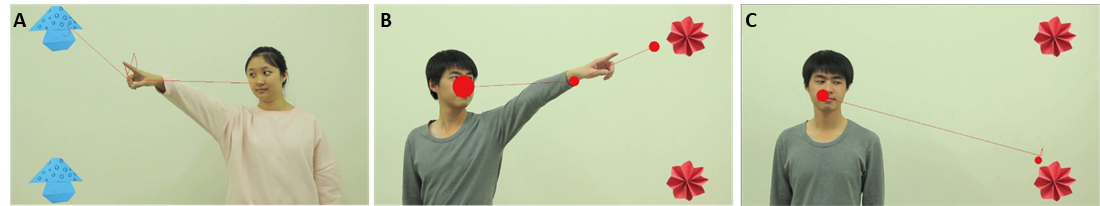


**Supplementary Figure 6.** Examples of three different types of RJA responses to video clips identified by researchers and RJAfinder. **(A)** The participant showed obvious saccade and it was difficult to identify the existence of the real RJA events by researchers’ observations. **(B)** The position of the fixations exceeded a radius of 1.2 times the radius of the target object AOI. **(C)** The fixation returned to the actor in the fixation trajectory.

**
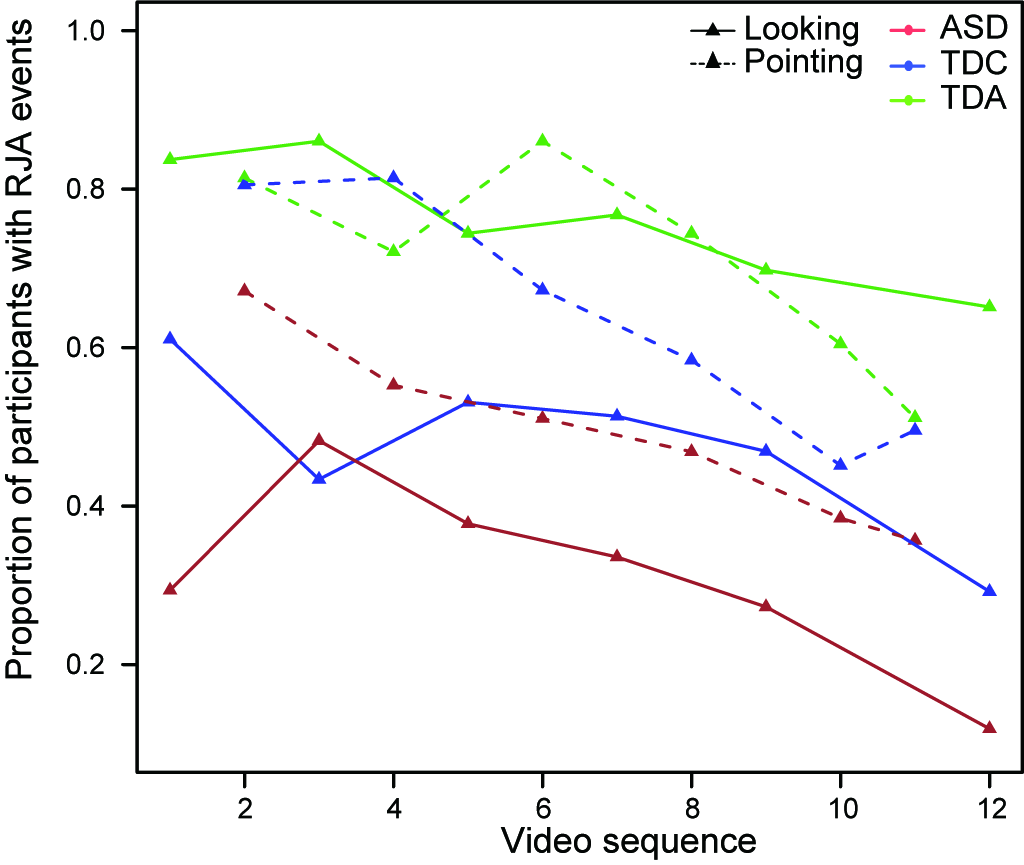
**

**Supplementary Figure 7.** Proportion of participants displaying RJA. All three groups displayed a lower RJA in response to looking stimuli than to pointing stimuli.


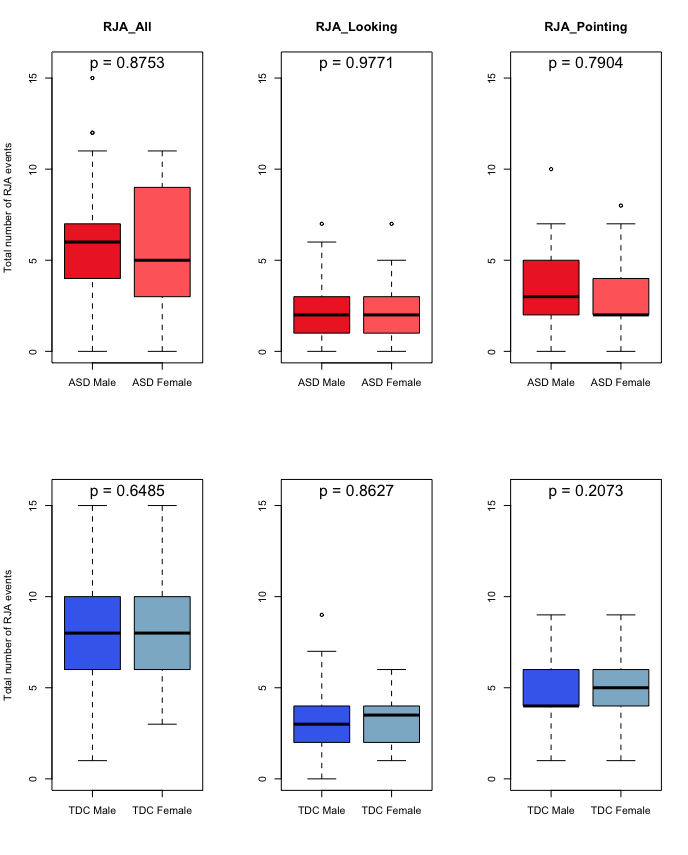


**Supplementary Figure 8.** RJA events detected in ASD and TDC participants of different genders in response to looking, pointing, and both stimuli combined. There was no statistically significant difference between genders among the ASD or TDC groups.


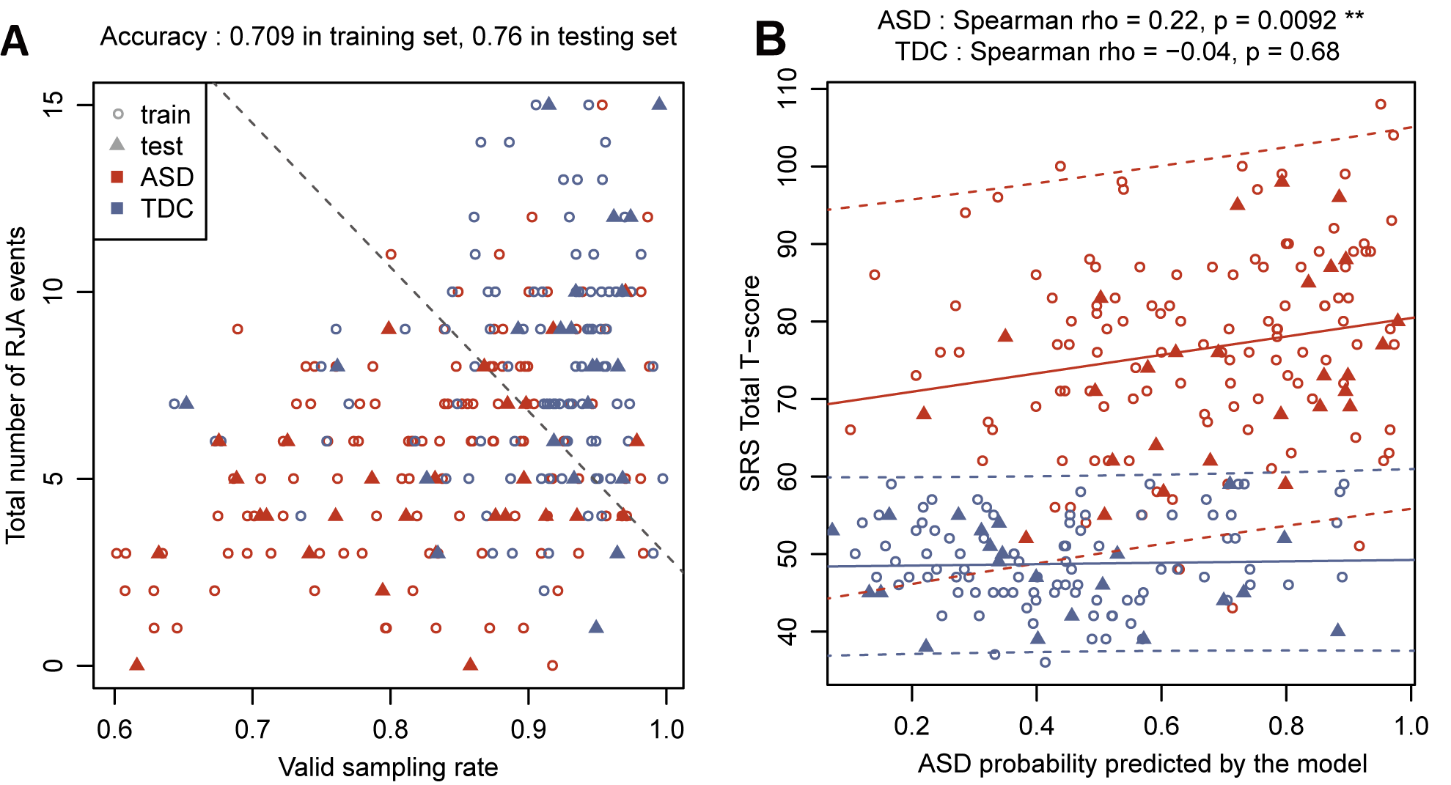


**Supplementary Figure 9. (A)** The average valid sampling rate and the total number of RJA events were used as two predictive variables. **(B)** Positive correlation of SRS total T-score and the ASD probability predicted by the logistic regression model.

**Supplementary Tables**

**Supplementary Table 1.** Raw eye tracking data exported from the Tobii Studio software.

| **Raw Data Matrix** | **Definition (From Tobii Studio)** | **Format** |
| --- | --- | --- |
| RecordingName | Name of the recording |  |
| RecordingTimestamp | Timestamp at the start of recording (t0 = 0 ms) |  |
| StudioEvent | Type of media element or manual logging event (START, END, and manual logging description) | Milliseconds |
| FixationIndex | Position in a series of fixations and saccades. The index is an auto-increment number starting with 1 (first fixation detected). | Count |
| GazeEventDuration | Duration of an eye movement event. | Milliseconds |
| FixationPointX (MCSpx) | Horizontal coordinate of the fixation point on the screen. This column is affected by the settings in the Fixation Filter Tab (Global Settings) | Pixels |
| FixationPointY (MCSpx) | Vertical coordinate of the fixation point on the screen. This column is affected by the settings in the Fixation Filter Tab (Global Settings) | Pixels |
| AOI[Name of AOI]Hit | Reports whether the area of interest (AOI) is active and whether the fixation is located inside of the AOI: Empty = the media on which the AOI is positioned is inactive; -1 = AOI not active; 0 = AOI active and the fixation is not focused on the AOI; 1= AOI active and the fixation is focused on the AOI. Enabling this column generates one column per AOI. | Empty;-1;0;1 |

**Supplementary Table 2.** The interpretation of the Cohen’s kappa values.

| Kappa values | Interpretation |
| --- | --- |
| < 0 | Poor agreement |
| 0.0 ~ 0.20 | Slight agreement |
| 0.21 ~ 0.40 | Fair agreement |
| 0.41 ~ 0.60 | Moderate agreement |
| 0.61 ~ 0.80 | Substantial agreement |
| 0.81 ~ 1.00 | Almost perfect agreement |

**Supplementary Table 3.** Correlation of SRS T-scores with the total number of RJA events.

| SRS Items | ASD (n = 143) | | | TDC (n = 113) | | |
| --- | --- | --- | --- | --- | --- | --- |
|  | Rho | Uncorrected  *p*-value^a^ | BH^b^ corrected  *p*-value | Rho | Uncorrected  *p*-value^a^ | BH^b^ corrected  *p*-value |
| Total T-scores | -0.15 | 0.078 | 0.156 | 0.120 | 0.20 | 0.392 |
| Social awareness | -0.050 | 0.55 | 0.551 | 0.056 | 0.56 | 0.600 |
| Social cognition | -0.18 | 0.033* | 0.156 | 0.11 | 0.26 | 0.392 |
| Social communication | -0.15 | 0.077 | 0.156 | 0.13 | 0.17 | 0.392 |
| Social motivation | -0.12 | 0.15 | 0.223 | 0.17 | 0.067 | 0.392 |
| Autistic mannerisms | -0.094 | 0.27 | 0.320 | -0.050 | 0.60 | 0.600 |

^a^Spearman correlation * indicates significant differences (*p* < 0.05)

^b^BH: Benjamini-Hochberg procedure

**Supplementary Table 4.** The group difference and effect size for comparison of RJA ability among three groups.

|  |  |  | ***p*-value** | **effect size**  **(r value)** | **effect size interpretation** | **power** |
| --- | --- | --- | --- | --- | --- | --- |
| **FIGURE 2A.** The proportion of participants having RJA events in the three groups for the 12 clips |  | ASD vs TDC | 2.4×10^-2^ | 0.46 | medium | 0.527 |
|  |  | TDC vs TDA | 7.2×10^-3^ | 0.55 | large | 0.978 |
|  |  | ASD vs TDA | 9.67×10^-5^ | 0.80 | large | 1 |
| **FIGURE 3A.** The valid sampling rate of the three groups for 12 clips |  | ASD vs TDC | 1.95×10^-11^ | 0.42 | medium | 1 |
|  |  | TDC vs TDA | 1.93×10^-4^ | 0.30 | small | 0.919 |
|  |  | ASD vs TDA | 6.17×10^-14^ | 0.55 | large | 1 |
| **FIGURE 3B.** Total number of RJA events observed in the three groups for 12 clips | All | ASD vs TDC | 4.91×10^-10^ | 0.39 | medium | 0.999 |
|  |  | TDC vs TDA | 1.84×10^-5^ | 0.34 | medium | 0.928 |
|  |  | ASD vs TDA | 2.87×10^-12^ | 0.51 | large | 1 |
|  | Looking | ASD vs TDC | 1.05×10^-6^ | 0.31 | medium | 0.987 |
|  |  | TDC vs TDA | 3.14×10^-9^ | 0.47 | medium | 1 |
|  |  | ASD vs TDA | 3.43×10^-15^ | 0.58 | large | 1 |
|  | Pointing | ASD vs TDC | 2.06×10^-8^ | 0.35 | medium | 0.999 |
|  |  | TDC vs TDA | 3.46×10^-1^ | 0.08 | small | 0.042 |
|  |  | ASD vs TDA | 8.18×10^-6^ | 0.33 | medium | 0.959 |

**Supplementary Table 5.** Coefficients of the logistic regression model.

|  | estimate | std error | | *p*-value |
| --- | --- | --- | --- | --- |
| Intercept | 8.94 | | 1.87 | 1.74×10^-6^ |
| The average valid sampling rate | -8.30 | | 2.15 | 1.10×10^-4^ |
| The total number of RJA events | -0.22 | | 0.06 | 2.83×10^-4^ |

**Supplementary Table 6.** 10-fold cross-validation of the logistic regression model.

|  | **CV_mean** | **CV_sd** |
| --- | --- | --- |
| k | 5.50 | 3.03 |
| AUC | 0.79 | 0.14 |
| accuracy | 0.74 | 0.12 |
| sensitivity | 0.80 | 0.18 |
| specificity | 0.69 | 0.17 |
| precision | 0.75 | 0.14 |
| FDR | 0.25 | 0.14 |

**Supplementary Table 7.** The correlations of SRS T-scores with the predicted ASD probability by the logistic regression model.

| SRS Items | All data | | | ASD | | | TDC | | |
| --- | --- | --- | --- | --- | --- | --- | --- | --- | --- |
|  | Rho | Uncorrected  *p*-value | BH^b^ corrected  *p*-value | Rho | Uncorrected  *p*-value | BH^b^ corrected  *p*-value | Rho | Uncorrected  *p*-value | BH^b^ corrected  *p*-value |
| Total T-scores | 0.47 | 3.54×10^-15^ | 1.06×10^-14^ | 0.22 | 0.0092 | 0.0276 | -0.04 | 0.68 | 0.83 |
| Social awareness | 0.43 | 3.54×10^-13^ | 5.31×10^-13^ | 0.14 | 0.098 | 0.0980 | 0.02 | 0.83 | 0.83 |
| Social cognition | 0.48 | 7.40×10^-16^ | 4.44×10^-15^ | 0.25 | 0.0030 | 0.0180 | -0.03 | 0.73 | 0.83 |
| Social communication | 0.46 | 1.31×10^-14^ | 2.62×10^-14^ | 0.19 | 0.022 | 0.0440 | -0.06 | 0.53 | 0.83 |
| Social motivation | 0.38 | 5.39×10^-10^ | 5.39×10^-10^ | 0.16 | 0.056 | 0.0840 | -0.13 | 0.185 | 0.83 |
| Autistic mannerisms | 0.40 | 3.68×10^-11^ | 4.42×10^-11^ | 0.15 | 0.078 | 0.0936 | 0.07 | 0.45 | 0.83 |

^b^BH: Benjamini-Hochberg procedure
